# Clinical and Radiographic Evaluation of Four Different Pulpotomy Agents in Primary Molars: A Longitudinal Study

**DOI:** 10.5005/jp-journals-10005-1443

**Published:** 2017-02-27

**Authors:** B Sunitha, Ravindar Puppala, Balaji Kethineni, Manoj K Mallela, Ravigna Peddi, P Tarasingh

**Affiliations:** 1 Assistant Professor, Department of Pedodontics and Preventive Dentistry, Sri Venkata Sai Institute of Dental Sciences, Mahbubnagar Telangana, India; 2 Professor and Head, Department of Pedodontics and Preventive Dentistry, Sri Venkata Sai Institute of Dental Sciences, Mahbubnagar Telangana, India; 3 Professor, Department of Pedodontics and Preventive Dentistry, Sri Venkata Sai Institute of Dental Sciences, Mahbubnagar Telangana, India; 4 Professor, Department of Pedodontics and Preventive Dentistry, Sri Venkata Sai Institute of Dental Sciences, Mahbubnagar Telangana, India; 5 Associate Professor, Department of Pedodontics and Preventive Dentistry, Sri Venkata Sai Institute of Dental Sciences, Mahbubnagar Telangana, India; 6 Assistant Professor, Department of Pedodontics and Preventive Dentistry Government Dental College, Hyderabad, Telangana, India

**Keywords:** Emdogain, Mineral trioxide aggregate, Pulpotec, Pulpotomy.

## Abstract

**Background:**

The medicament formocresol (FC) used for pulpotomy in primary teeth has great concerns regarding its toxicity due to one of its constituent formaldehyde which acts by tissue fixation. Therefore, new medicaments were introduced which claimed preservation and regeneration of pulp.

**Aim:**

The present study is aimed to compare and evaluate the clinical and radiographic success of FC, pulpotec, mineral trioxide aggregate (MTA), and emdogain (EMD) as pulpotomy medicaments in human primary molars.

**Design:**

A sample of 21 patients with 84 teeth were selected. All the patients have at least four teeth eligible for pulpotomy according to selection criteria. In each mouth, the teeth selected were randomly allocated into four groups with 21 each.

**Results:**

After 24 months of follow-up, the clinical success rates were FC (94%), pulpotec (94%), MTA (100%), and EMD (83%) and radiographically FC (88%), pulpotec (83%), MTA (94%), and EMD (72%), which were statistically not significant (p > 0.05).

**Conclusion:**

The outcome of this study demonstrates MTA has a high success rate compared with FC, pulpotec, and EMD as pulpotomy agent. In addition, MTA, pulpotec, and EMD can be considered as alternatives to FC as pulpotomy agent.

**How to cite this article:**

Sunitha B, Puppala R, Kethineni B, Mallela MK, Peddi R, Tarasingh P. Clinical and Radiographic Evaluation of Four Different Pulpotomy Agents in Primary Molars: A Longitudinal Study. Int J Clin Pediatr Dent 2017;10(3):240-244.

## INTRODUCTION

Pediatric endodontics, an ever-advancing field, always needs to evaluate the existing and upcoming materials to find better biologic alternatives. Thus the future clinicians will have at their disposal novel therapeutic approaches for children and adolescents. Vital pulpot-omy process using FC has been in practice since decades due to its simplicity and good prognosis.^1^ However, many studies showed concern about the use of FC in humans due to its toxicity and the International Agency for Research on Cancer has classified formaldehyde as carcinogenic to human beings.^2^ This raised a need for an alternate pulpotomy medicament.^3^ Since then, a wide range of materials have been used over the years to replace FC.

Pulpotec is a medicament to perform pulpotomy in deciduous molars which is a radiopaque, nonresorb-able paste. Its powder consists of polyoxymethylene, iodoform, and zinc, and liquid consists of dexametha-sone, formaldehyde, phenol, guaiacol. Many clinical trials have shown that pulpotec by its antiseptic, hemostatic, anesthetic, antimicrobial, and antiinflam-matory actions and with the advantage of having clinical success even in cases with a little residual blood in the pulp chamber can be used as a pulpotomy medicament.^4-6^

Mineral trioxide aggregate (MTA) was demonstrated as a successful pulpotomy medicament and as an appropriate alternative to FC because it consistently showed better results both clinically and radio-graphically.^7-10^ Recently, EMD has been introduced as a pulpotomy material. Enamel matrix proteins, mainly "amelogenins," are secreted by Hertwig’s epithelial root sheath during tooth development and induce a cellular cementum formation, capable of stimulating periodontal ligament cell proliferation sooner than gingival fibroblasts and bone cells.^11^ In animal studies EMD has been successfully used as a pulpotomy agent in noninfected teeth.^12^ Literature shows comparative studies between the medicaments FC *vs* EMD, FC *vs* MTA, MTA *vs* EMD. But comparative studies using all four medicaments in randomized clinical trial are not available. Therefore, the uniqueness of the present study is FC, pulpotec, MTA, and emdogain, which were used as a pulpotomy medicament in the primary molars and its efficacy was observed both clinically and radiographically over a period of 2 years.

## MATERIALS AND METHODS

A total of 21 children (8 boys and 13 girls) were selected among patients attending the Pedodontics Dental Clinic of Sri Venkat Sai Institute of Dental Sciences, Mahbub-nagar in Telangana state of India. Children who are cooperative with good general health and had at least four primary molars requiring pulpotomy were selected. A sample of 84 teeth was randomly allocated into four groups: Group I (FC), Group II (pulpotec), Group III MTA, and Group IV EMD with 21 teeth to each group as shown in Tab1e 1 (sample distribution). Teeth were selected according to the following criteria: Presence of deep carious lesions (radiographically, approximating the pulp); large pulp exposure during caries excavation. The study was conducted from November 2012 to March 2015.

The ethical clearance was obtained from the Institutional Ethical Committee. An informed consent was taken from the parent prior to the procedure. After profound local anesthesia, the tooth was isolated with a rubber dam. Removal of caries was done using sterile spoon excavator and roof of the pulp chamber was removed with a high-speed no. 330 sterile bur. The coronal pulp tissue was amputated by using a sterile sharp spoon excavator. Post amputation bleeding was controlled by placing sterile saline wet cotton pellets over radicular pulp stumps, waited for 5 minutes for hemostasis. If hemorrhage continued, the patient was excluded from the study and treated with appropriate treatment. Pulp dressings were applied as follows:

- *Group I (FC group):* A sterile cotton pellet moistened with FC (diluted at 5:1 ratio) was placed on the amputated pulp for 5 minutes, and pulp stumps were covered with zinc oxide eugenol cement, above which intermediate restorative material (IRM) was placed as temporary restoration.
- *Group II (pulpotec group):* The pulp stumps were covered with pulpotec paste (Products Dentaire - PD, Switzerland) comprised of powder and liquid mixed, above which IRM was placed.
- *Group III (MTA group):* The pulp stumps were covered with an MTA paste comprised of MTA powder (ProRoot MTA; Dentsply Tulsa Dental, Tulsa, OK) mixed at a 3:1 ratio with sterile saline, above which IRM was placed as temporary restoration.
- *Group IV (EMD group):* The canal orifices were dressed with EMD gel (Straumann Basel, Switzerland) above which the IRM was placed as a temporary restoration.

Final restorations were performed with stainless steel crowns. The patients were recalled for clinical and radiographic examinations at 6 months interval for a follow-up period of 2 years.^13,14^ Teeth were considered to be successful clinically when there is absence of pain, swelling or abscess, sinus tract opening, mobility, pain on percussion and radiographic absence of pathological root resorption, widening of periodontal space, bifurcation radiolucency, and periapical radiolucency.

A sample of 18 children (8 boys and 10 girls) and 72 teeth were available for follow-up evaluations. (Three children did not come for follow-up appointment at 6 months and were excluded from study.) Statistical analysis of the differences in treatment outcomes was performed by using the Chi-square test.

**Table 1:** **Table 1:** Evaluation of clinical failures

		*No pathology*		*Presence of pain*		*Swelling*		*Sinus tract opening*		*Presence of mobility*		*Total*	
Formocresol		17		–		1		–		–		18	
Pulpotec		17		–		1		–		–		18	
Mineral trioxide aggregate		18		–		–		–		–		18	
Emdogain		15		–		1		2		–		18	

## RESULTS

The treatment results for the four groups at each follow-up periods are presented in Flow Chart 1. Radiographic failures were significantly higher than the clinical failures (p < 0.05). At the 6th month follow-up examination, all teeth were rated successful both clinically and radiographically. At 12 months follow-up, two failures were observed in FC and pulpotec groups, one in MTA group and four failures in EMD groups. At 18 months follow-up, one failure was observed in pulpotec group and no failures in the other three groups. At 24 months follow-up, one additional failure was seen in EMD group. The overall failures observed were two in FC group, three in pulpotec group, one in MTA group, and five in EMD group.

**Graph 1:**
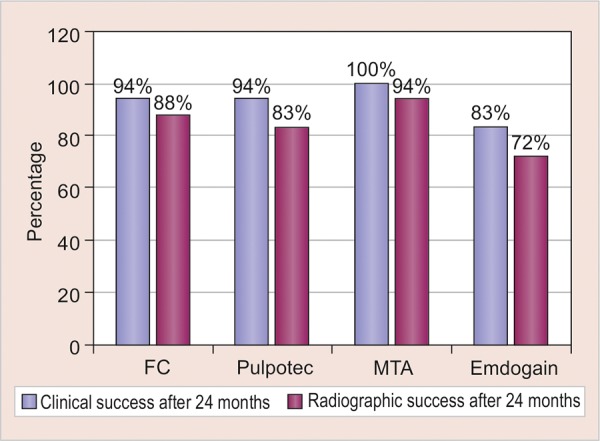
Comparison of overall success rates of four groups both clinically and radiographically after 24 months

By the end of 24 months follow-up, the overall failure clinically was five. One in FC group, one in pulpotec group and three in EMD, of which one showed swelling and two showed sinus tract (Table 1). Radiographic appearance of the pulp in the 72 pulpotomized teeth is shown in Table 2. The most frequently observed radio-graphic finding was external resorption followed by pulp canal obliteration and internal resorption. The clinical success rates of FC and pulpotec were 94.4%, EMD was 83.3%, and MTA was 100% (Table 3). Whereas the overall success rates (as shown in Table 4 and Graph 1) were 88.8% for FC, 83.3% for pulpotec, 94.4% for MTA and 72% for EMD, which was statistically not significant (p > 0.05).

**Flow Chart 1:**
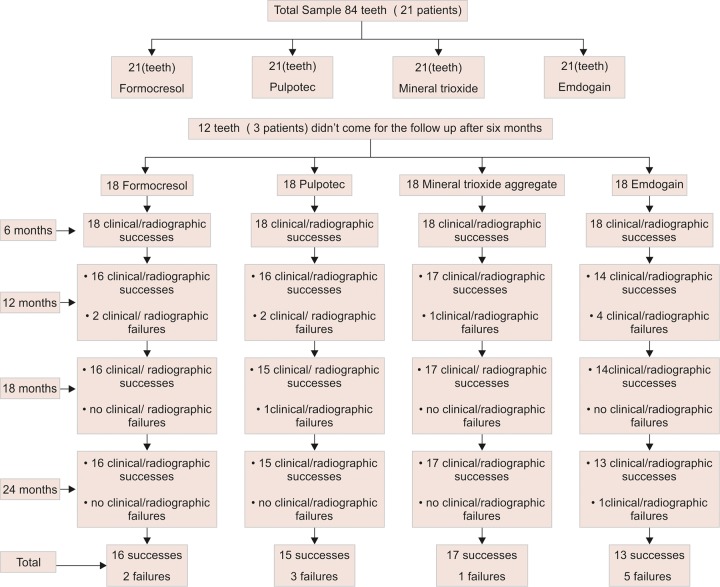
Sample follow-up for 2 years of period

**Table 2:** **Table 2:** Evaluation of radiographic failures

		*No* *pathology*		*Pulp canal* *obliteration*		*Internal* *resorption*		*External* *resorption*		*Total*	
FC		16		1		–		2		18	
Pulpotec		15		3		1		3		18	
MTA		17		1		–		1		18	
Emdogain		14		4		2		4		18	

**Table 3:** **Table 3:** Clinical evaluation of four pulpotomy groups during follow-up period for 2 years

*Groups*		*6 months*		*12 months*		*18 months*		*24 months p-value*			
FC		18 (100)		18 (100)		17 (94)		17 (94)		NS	
Pulpotec		18 (100)		18 (100)		17 (94)		17 (94)		NS	
MTA		18 (100)		18 (100)		18 (100)		18 (100)		NS	
EMD		18 (100)		17 (94)		15 (83)		15 (83)		NS	

Values given in parenthesis are success percentage; p value is NS: Not significant

## DISCUSSION

Pulpotec has an advantage of having clinical success even in cases with a little residual blood in the pulp chamber when used as a pulpotomy medicament.^5,6^ The results of pulpotec are in agreement with Donskaya and Dedeyan^5^ in their clinical trials using pulpotec as pulpotomy medicament and reported easiness, simplicity in using pulpotec. The paste hardens quickly after mixing of ingredients, providing optimal conditions for final restoration with its added hemostatic and fixative properties.^4-6^ However, in our study, pulpotec application was simple, time-saving, and affordable.

A study conducted by Holan et al^9^ comparing the clinical and radiographic success rates between FC and MTA pulpotomy in primary molars showed success rates of 97% for MTA and 83% for FC. Aeinehchi et al^7^ conducted a randomized control trial of MTA and FC for pulpotomy in primary teeth and found higher success rates of MTA when compared with FC. A similar result was seen in our study with 94% success rate of MTA.

**Table 4:** **Table 4:** Radiographic evaluation of four pulpotomy groups during follow-up period for 2 years

*Groups*		*6 months*		*12 months*		*18 months*		*24 months*		*p-value*	
FC		18 (100)		16 (88.8)		16 (88.8)		16 (88.8)		NS	
Pulpotec		18 (100)		16 (88.8)		15 (83)		15 (83)		NS	
MTA		18 (100)		17 (94)		17 (94)		17 (94)		NS	
EMD		18 (100)		14 (77.7)		14 (77.7)		13 (72)		NS	

Values given in parenthesis are success percentage; p-value is NS: Not significant

This may be due to its property of becoming hard in the presence of moisture, which in turn evoked a better sealing of pulp chamber. Therefore, MTA can be used in areas in which it is virtually impossible to achieve a totally dry environment, and MTA is a promising material with an expanding foundation of research. It is gaining interest as pulpotomy medicament because of its excellent sealability, biocompatibility, and dentinogenic properties.

The EMD derivative obtained from developing porcine teeth has been approved by the US Food and Drug Administration and marketed under the trade name "EMDOGAIN." It has been successfully used as a pulpotomy agent in noninfected teeth in animal studies. This material is a viscous gel consisting of enamel-derived proteins in a polypropylene liquid and of 90% amelo-genins, rest are proline-rich nonamelogenins, tuftelin, tuft protein, serum proteins, ameloblastin, and amelin.^13,14^

The common radiographic failure in the present study (Table 5) was interradicular radiolucency and internal resorption found in 4 out of 21 cases in EMD group during 6 to 12-month interval. In the EMD group, the clinical follow-up at the 6th month revealed no signs of clinical failure. However, at the end of 12th month, one tooth reported with swelling, two teeth showed sinus tract opening, and the same teeth showed furcation radiolu-cency. These three teeth were treated with pulpectomy. The clinical success with EMD at 24 months was 93.3% and radiographic success rate was 73.3%. The findings were similar to that of Sabbarini et al.^15^

The higher failure rate in EMD might be due to handling properties of the material which is available in a gel form and standardization of the quantity, which was difficult during application over the vital pulp tissue. Another disadvantage with EMD material is that care should be taken to prevent the gel to flow away from the amputation site while placing of temporary material (IRM). However, a significant disadvantage was the entire material has to be used up within 2 hours or the material loses its efficacy. Remaining quantity was discarded if not used in the proper time, which greatly reduces the cost-effectiveness of the material. The clinical and radiographic assessment of EMD pulpotomized teeth in this study offers evidence that EMD is a promising material that may be as good as other pulpotomy agents. Further refinement of the delivery system is needed to ensure that EMD can be developed as an agent for predictable and cost-effective in preservation of vitality of radicular pulp.

**Table 5:** **Table 5:** Distribution of the sample

		*Formocresol*						*Pulpotec*						*Mineral trioxide aggregate*						*Emdogain*					
*Evaluation intervals (months)*		*Total teeth*		*Lower primary first molars*		*Lower primary second molar*		*Total teeth*		*Lower primary first molars*		*Lower primary second molar*		*Total teeth*		*Lower primary first molars*		*Lower primary second molar*	
3		21		10		11		21		10		11		21		10		11		21		11		10	
6		18		9		9		18		8		10		18		8		10		18		10		8	
12		18		9		9		18		8		10		18		8		10		18		10		8	
18		18		9		9		18		8		10		18		8		10		18		10		8	
24		18		9		9		18		8		10		18		8		10		18		10		8	

Failure of pulpotomy in primary molars was attributed to several factors, one of which was erroneous diagnosis of a chronically inflamed pulp as noninflamed and nonin-fected.^16^ Pulpotomy agents that require a healing process may not be applicable to all cariously involved primary teeth. More accurate diagnostic tests may be required to choose those teeth with healthy radicular pulps. Many alternatives have been proposed to maintain the radicular pulp vitality among which MTA and pulpotec are gaining interest in recent years. The regenerative potential of EMD has also proved to be advantageous in vital pulp therapy procedures of primary teeth with encouraging results, although based on the few supporting studies. In the light of present findings of this study as well as documentary evidence, it appears that Pulpotec, MTA, and EMD can be acceptable alternatives to conventional FC pulpotomy procedure. Overall, MTA showed better success rate (94.4%) than pulpotec group (83.3%) and EMD group (72%). However, there is a need for further research to conclude the treatment outcome with histological collaboration, until tooth exfoliation.

## CONCLUSION

In cariously exposed primary molars, MTA could be used as a safe medicament for pulpotomy and could be a substitute for FC. Pulpotec and EMD used for covering the radicular pulp remnant after pulpotomy was proven to be promising alternatives as a pulp medicament. The study highlights on the biological agents like MTA and EMD that were useful for pulpotomy over formaldehyde.
